# Team-based vs. problem-based learning in undergraduate surgery: a randomized controlled trial in Saudi Arabia

**DOI:** 10.3389/fmed.2026.1767370

**Published:** 2026-02-09

**Authors:** Mohammad Shawir, Ehab A. Frah, Shadad M. Mahmoud, Marai Mohammed Alamri, Yazeed Ibrahim Alghabban, Mohammed M. Aljohani, Roaa Ghazi Khan

**Affiliations:** 1 Department of Surgery, Faculty of Medicine, University of Tabuk, Tabuk, Saudi Arabia; 2 Department of Statistics, Faculty of science, University of Tabuk, Tabuk, Saudi Arabia

**Keywords:** team-based learning, problem-based learning, undergraduate surgery, medical education, Saudi vision 2030

## Abstract

**Objectives:**

The primary objective measured in our study is to determine whether Team-Based Learning (TBL) is a superior pedagogical approach compared to Problem-Based Learning (PBL) or not. We focused on our secondary objectives, which include promoting problem-solving, facilitating independent learning, and engaging students. The study was conducted among sixth-year clinical students at the Faculty of Medicine, University of Tabuk.

**Methods:**

In this interventional study, we targeted 66 medical students in the final clinical year in our faculty of medicine at the University of Tabuk during the period from 15/10/2024 to 15/01/2025. The control group (PBL) comprised 32 students, whereas the case group (TBL) comprised 34 students. At the end of the clinical sessions, all 66 students took a summative test to assess their knowledge using 14 multiple-choice questions (MCQs) that were part of an online self-administered, voluntary questionnaire. Independent-samples statistical tests were used to compare the outcomes of perception, skills, attitude, and practical competency scores. All analyses were performed using SPSS software (Version 27), and statistical significance was defined as two-tailed *p* < 0.05.

**Results:**

The study revealed that students in the TBL group achieved an average score of 9.94 out of 14 on MCQs, with a standard deviation of 2.28. In contrast, the PBL group had an average score of 8.84 and a standard deviation of 2.24, with a *p*-value of 0.053 for both groups. The cumulative knowledge derived from summative test results was not statistically significant for either group. In the (TBL) case-control experiment, the pre-class test mean score was 7.42 (*SD* = 2.50), while the post-test scores averaged 9.94 (*SD* = 2.35). This difference was statistically significant (*P* = 0.001). This demonstrates the effectiveness of the TBL pedagogical loop in enhancing knowledge acquisition during the learning experience.

**Conclusion:**

The TBL and PBL methodologies yielded comparable summative outcomes for both student cohorts. The systematic implementation of TBL as a pedagogical tool can enhance students’ knowledge acquisition. Our study found that final-year MBBS students prefer both PBL and TBL.

## 1 Introduction

Team-based learning (TBL) and problem-based learning (PBL) are two learning methodologies that focus on the development, execution, and completion of tasks and projects, respectively. While students form the core of both strategies’ learning, it’s crucial to remember that the establishment of objectives precedes their implementation. TBL and PBL are two separate methodologies that provide significant educational experiences for students. PBL emphasizes multidisciplinary project work that cultivates critical thinking, problem-solving, and collaborative abilities, while TBL focuses on language acquisition through the execution of specific tasks. Each method possesses distinct attributes, advantages, and factors for execution.

Eva Koritakova et al. (1) stated that both strategies, TBL and PBL, exhibit distinct advantages and disadvantages relative to one another (2). Theoretically, though, TBL offers the benefits of readiness assessment, expert availability, and the provision of introductory materials. “Learning gain was higher with TBL compared to PBL, with no difference in knowledge retention and the effect on different parameters of critical thinking skills,” according to Pakhmode et al. (3). Students perceived TBL as more advantageous for the undergraduate curriculum (3). According to Burgess et al. (4), “The students found positive aspects of their TBL experience to include the smaller group size, the use of readiness assurance tests, immediate feedback from senior clinicians, and time efficiency.” In PBL, students indicated that the varying skills of instructors, insufficient direction, and huge group size hampered their learning. “(4). TBL comprises six steps, including pre-class preparation, individual assurance test, team-based assurance test (pre-class test iRAT and tRAT), immediate feedback, clinical problem-solving discussion, and closure (4–6). It has been stated that, despite TBL’s increasing popularity, it did not lead to higher long-term retention (7). According to Nawabi et al. (8), “students reported positive aspects of the TBL experience, such as a more engaging format, collaborative learning, teamwork, and group competition.” They claimed that PBL had improved their research, presentation, and clinical reasoning abilities (8). However, PBL has the advantage of incorporating in-depth clinical reasoning into the problem-solving process. As a result, Annette Burgess et al. recommended a hybrid strategy that combines the benefits of both (4–6). Dolmans et al. (9) support a combined technique to maximize students’ learning outcomes (9). Research has demonstrated that TBL improves the performance of pharmacology students compared to those who do not attend. As a result, Nora et al. recommended the incorporation of TBL as a teaching component in the medical curriculum (10). Fujiwara et al. has proven that TBL, involving online physiology, is a useful method during epidemics such as COVID-19. It was understandable that in-person instruction was not an option. (11). Mulugeta and Zemedkun conducted a limited pilot cross-sectional study in Ethiopia, highlighting the inherent limitations of Team-Based Learning (TBL), specifically noting criticisms regarding the time required for TBL preparation and the necessity for student readiness (12). TBL effectively supplanted PBL in the first and second years of the medical curriculum, receiving favorable feedback from participants. The primary critique was inadequate facilitation by the expert, poor alignment of pre-class preparation with the Team-Based Learning (TBL) patient case, a lack of availability of the Team Readiness Assurance Test (TRAT), and excessive duration leading to fatigued students (13). Project-Based Learning (PBL) as a pedagogical approach did not yield superior information retention or academic achievement compared to traditional techniques (14). Zhao et al. have shown that “PBL combined with case-based learning (CBL) may be an effective approach for enhancing the performance and clinical skills of medical students and residents” (15). Despite their enjoyment of the activity, participants could not have a favorable opinion of the tutor’s post-PBL evaluation (16). Moreover, Saudi medical students favored problem-based learning (PBL) over lecture-based learning (LBL) due to PBL’s superior clinical reasoning capacity, clinical solving capabilities, and in-depth discussion (16). Nevertheless, there is less research comparing TBL to PBL. PBL with micro-video in burn instruction has outperformed LBL in terms of critical thinking and student satisfaction (17). “Online TBL has the potential to improve educational effectiveness for community pharmacy during the COVID-19 pandemic” (18) as opposed to PBL. Based on the findings of Sterpu et al., “TBL sessions may be advantageous in minimizing faculty workloads without compromising students’ learning outcomes” (19). “TBL resulted in improved attitudes toward obesity and self-perceived knowledge of obesity among first-year medical students at Case Western Reserve University School of Medicine (CWRU SOM),” reported Olson et al. (20). In summary, studies on TBL generally concur that positive medical attitudes and perceptions are prevalent. Nevertheless, the results of TBL-based knowledge retention and enhanced performance are contradictory (21). Our study aims to gain insight into whether TBS is a better teaching tool than PBL or not, keeping our objectives in mind.

The objective was to compare the PBL/TBL method concerning problem-solving, independent learning, engagement, and summative assessment outcomes.

## 2 Materials and methods

### 2.1 Inclusion criteria

All final clinical-year female MBBS students were selected as participants in the study, with around 66 individuals participating. The study included general surgery as a subject for final-year MBBS students at the University of Tabuk.

### 2.2 Exclusion criteria

The study encompassed all final clinical-year female MBBS students at the University of Tabuk. There exists a separate medical college branch for males and another for females, precluding the possibility of including both simultaneously. We have chosen to include solely the female cohort, as it was unfeasible to conduct a synchronous study involving male students due to time constraints, the segregation of the male cohort from the female cohort, and the author’s limited availability. This study excluded preclinical and paraclinical students due to their lack of patient interactions. Non-surgical subjects were excluded.

### 2.3 Trial design

This study tests the hypothesis that the two teaching methods produce different outcomes in student knowledge, attitudes, practices, and satisfaction with the instructional approach. This is an interventional study utilizing a randomly controlled trial. Our targeted population (66 medical students) were the final medical students in our faculty of medicine at the University of Tabuk during the period from 15/10/2024 to 15/01/2025. The control group (PBL) comprised 32 students, whereas the case group (TBL) comprised 34 students. At the end of the clinical sessions, all 66 students took a summative test to assess their knowledge using 14 multiple-choice questions (MCQs) that were part of an online self-administered, voluntary questionnaire, which was conducted in the presence of a clinical expert in the same examination room. All 66 students were double-blinded and randomized into groups (TBL 34 and PBL 32). In other words, neither of the students nor the clinical experts were aware of who joined which group, as all the students were Saudi females, putting on their religious/cultural face veils, thereby hiding their biometric identities. The case group in TBL 34 followed the usual teaching steps, which included pre-class materials, an individual assurance test (iRAT), a team-based assurance test (tRAT), immediate feedback, a discussion of clinical problems, and a wrap-up session. We randomly divided the case (TBL 34) group into seven groups in total, six groups consisting of five students, and the final group included four students. The exercise was supervised by an expert teacher with a facilitator in each group. The role of the facilitator was to lead the discussion in each group. The duration of each session was 4 h.

We provided each (TBL) group with pre-class preparatory material, which included recorded lectures on diabetic foot and abdominal hernias. The learning objectives were communicated to the students, and a pretest was performed at the start of the experiment. The case (TBL-34) group was provided the opportunity to read the subject before individually submitting themselves to an individual assurance test (iRAT) based on 14 MCQs—an online, self-administered, voluntary questionnaire. Thereafter, they discussed the 14 MCQs in the designated seven groups’ team-based assurance tests (tRAT). Following the previous step, they engaged in an intra-group immediate feedback reflection. The pedagogical loop concluded with a discussion on clinical problem-solving. In contrast, the control group (PBL32) was divided randomly into seven groups in total, with six groups comprising five each, and the last group had two students. The exercise was supervised by an expert teacher, with minimal interference if required, and a facilitator in each group. The role of the facilitator was to lead the discussion in each group. The duration of each session was 4 h.

**FIGURE 1:**
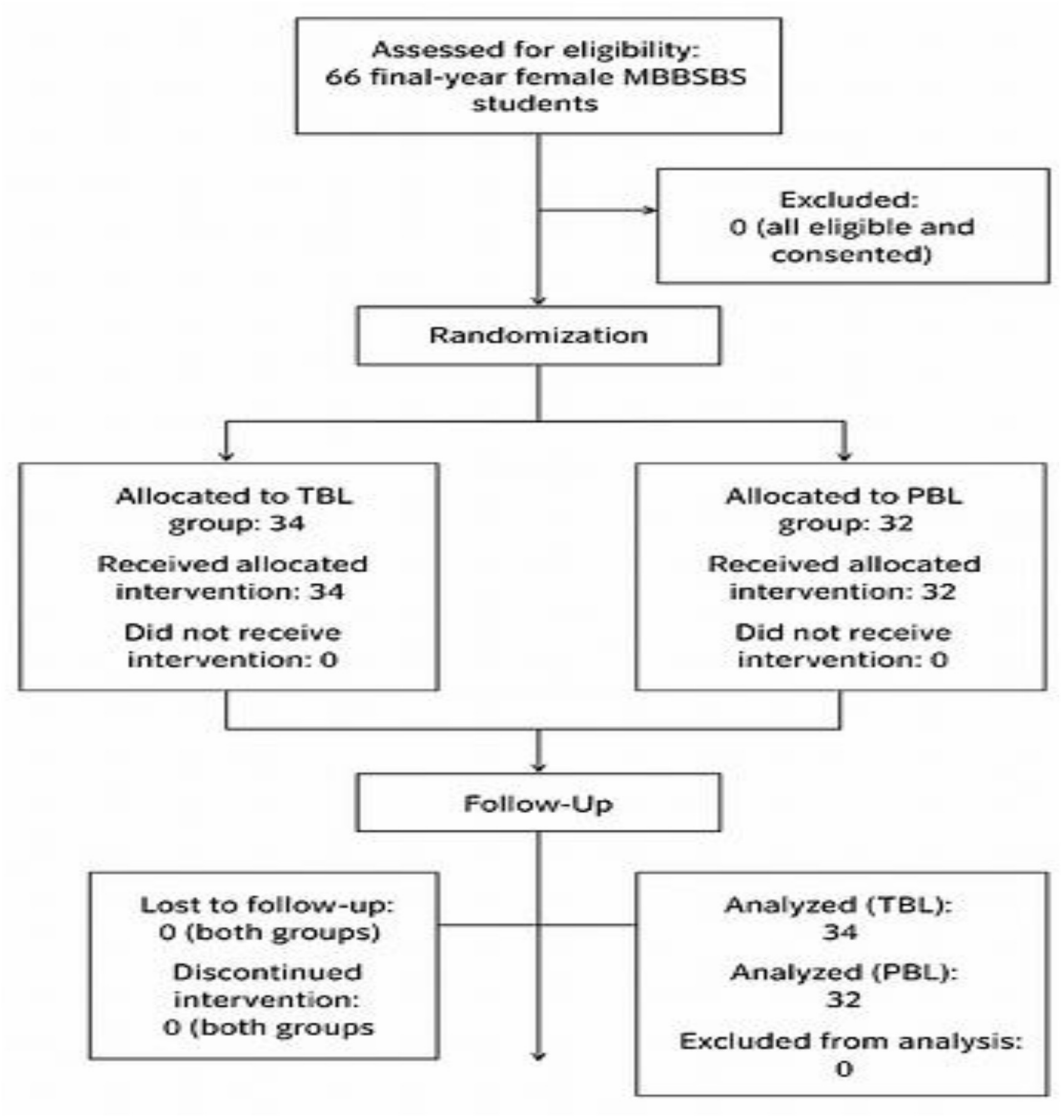
CONSORT flow diagram. CONSORT flow diagram of participant enrolment, randomization, allocation, follow-up, and analysis.

The control (PBL32) group did not have the privilege of pre-class material preparation. However, the summative test/assessment, which measured their knowledge, prompted them to engage in inert group discussions involving critical thinking and problem-based learning. At the end of the clinical experiment, both case groups (TBL34) and the control group (PBL32) completed an online questionnaire that they filled out themselves, which was optional and approved by TELSON, to share their thoughts and experiences about the experiment. We obtained the TELSON questionnaire with written permission from the original author, Eva Koritakova, via an official email. The questionnaire comprised 19 Likert scale questions, followed by four open-style questions. All student groups (TBL and PBL) completed the questionnaire on their iPhones and iPads, provided the internet connectivity was reliable (Figure 1).

### 2.4 Data acquisition

The pre-test, consisting of both the individual Readiness Assurance Test (iRAT) and the team Readiness Assurance Test (tRAT), was administered using Google Forms as a self-administered, voluntary online assessment. The iRAT was completed individually at the start of the session to evaluate baseline knowledge acquisition, followed immediately by the tRAT, which was completed collaboratively in assigned groups to support peer discussion and collective reasoning.

Learners’ perceptions of (TBL) and (PBL) were evaluated using the TELSON Learner Questionnaire. To facilitate structured statistical analysis, the questionnaire was categorized into three domains: Perception (6 items), Skills (7 items), and Attitude (6 items). A five-point Likert scale was used to rate each item, with 1 = strongly disagree, 2 = disagree, 3 = neutral, 4 = agree, and 5 = strongly agree. This allowed for a quantitative comparison of student responses across domains. The TELSON Questionnaire consists of three domains: Perception, Skills, and Attitude.

Scoring for the iRAT was based on the total number of correct responses completed individually out of 14 multiple-choice questions on diabetic foot and hernia. In the tRAT, student groups were given different questions on the same topic and scored by consensus. Group performance in the tRAT was compared with corresponding individual iRAT scores to evaluate the contribution of collaborative learning and group discussion to overall knowledge performance. The quantitative survey responses were analyzed using descriptive and inferential statistics.

Data were collected using structured electronic questionnaires administered through Google Forms for each study group. The questionnaire included predefined response fields to ensure consistency and facilitate standardized data entry. Following collection, all responses were exported to Microsoft Excel for data management. The dataset underwent an initial screening to verify completeness, during which records were examined for missing or inconsistent responses. Entries with substantial missing data were flagged for exclusion. The research team subsequently reviewed and cleaned the dataset to correct identifiable entry errors, remove duplicated responses, and ensure the accuracy of variable coding. Data integrity, internal consistency, and readiness for analysis were confirmed before proceeding to statistical processing.

### 2.5 Statistical analysis

The quantitative survey responses were analyzed using descriptive and inferential statistics. Qualitative questionnaire data were summarized using frequency distributions and crosstabulations. Between-group comparisons of perception, skills, attitude, and practical competency scores were conducted using independent-samples statistical tests. Summative assessment results were also evaluated using independent-samples procedures. All analyses were performed using SPSS software (Version 27), and statistical significance was defined as two-tailed *p* < 0.05. All three domains (Knowledge, Attitude, and Practice) demonstrate good internal consistency (α = 0.86–0.88), exceeding the acceptable threshold of 0.70 for medical education research. The overall questionnaire shows excellent reliability (α = 0.95), indicating that the instrument is highly reliable and consistent for measuring KAP among medical students/professionals. The questionnaire is psychometrically sound and suitable for use in medical education KAP studies. No items need removal; the instrument can be confidently applied for data collection and analysis.

### 2.6 Ethical approval

The Ethical Review Committee for Medical Research Involving Human Subjects at the University of Tabuk approved this study (approval number: UT-448-251-2024) on November 3, 2024. Participant responses were collected anonymously, and students were informed online that their completion of the survey constituted consent to participate in the research study, and all anonymized data were maintained. We clarified that survey participants would not incur any disadvantage for opting out of participation and that any disclosed data, even with consent, would omit personally identifiable information. Furthermore, we verified that the free text section of the questionnaire contained no personally identifiable information.

## 3 Results

### 3.1 Learners’ survey demographic results

Sixty-six female students voluntarily joined our study (*n* = 66). In the case group (TBL 34), all the students voluntarily joined the study with 100% enrolment and answered all 14 MCQs (summative assessments). Their questionnaires were examined for completeness, accuracy, and validity. The mean age for the case group (TBL) was 23 years old. The lowest age was 22 years old, and the highest was 26. In the case group (TBL), 91.7% reported prior experience with TBL, while 8.3% indicated no prior experience. The mean GPA for both the groups (TBL and BPL) was 4.06 with a standard deviation of 0.073 out of 62/66 students. Four students did not answer the question on their GPA. In the control group (PBL 32), all the students joined the study with a 100% enrolment rate and answered all 14 MCQs (summative assessments). Their questionnaires were examined for completeness, accuracy, and validity. The mode age for the control group (PBL 32) was 23 years old. The lowest age was 22 years old, and the highest was 26 years old. In the control group (PBL), all the students had previous experience with PBL, with a 100% response (Table 1).

**TABLE 1:** Baseline characteristics of participants.

Characteristic	TBL group (*n* = 34)	PBL group (*n* = 32)	Total (*N* = 66)
Age, mean (range)	23 (22–26)	23 (22–26)	23 (22–26)
GPA, mean ± SD	4.07 ± 0.58	4.07 ± 0.58	4.07 ± 0.58
Prior TBL exposure (%)	91.7%	–	–
Prior PBL exposure (%)	–	100%	–

Age and GPA are reported as mean values with range or standard deviation. Prior exposure percentages reflect self-reported experience with each method.

### 3.2 Results from Likert scale questions and additional feedback data

#### 3.2.1 Perception

Skill The students’ perception of applying their existing knowledge did not differ between the case group (TBL) at 83% and the control group (PBL) at 81%, respectively. Both student groups similarly believed that the two pedagogical strategies facilitated their engagement in formulating a reasonable differential diagnosis. The case group (TBL) students assessed the virtual scenario as appropriately challenging for their training, assigning it a rating of 77%, whereas the control group (PBL) rated it at 80%. Most of the students in the control groups (PBL), 85%, strongly agreed that they can arrive at a reasonable diagnosis in a real situation; in contrast to the case group (TBL), they were less certain at 78%. A 84% of the case group students (TBL) strongly agreed that the iRAT allowed them to self-assess and better understand which area they were strongest in, besides giving them the chance to discuss and justify their answers. In conclusion, both student groups (TBL and PBL) believed that both pedagogical strategies helped them in gaining their knowledge despite no statistical significance. The *P*-values were found to be 0.348 (Table 2).

**TABLE 2:** TELSON questionnaire domains.

Domain	TBL Mean ± SD	PBL Mean ± SD	*p*-value
Perception	4.05 ± 0.62	4.25 ± 0.58	0.348
Attitude	3.86 ± 0.71	4.21 ± 0.63	0.122
Skills	3.99 ± 0.66	4.10 ± 0.60	0.595

Comparison of TELSON domain scores between groups using independent-samples *t*-tests. No statistically significant differences observed.

#### 3.2.2 Skills

A 87% of the students in the control group (PBL) strongly agreed that it provoked high-quality discussion, while 85% of the case group (TBL) agreed. Both groups were satisfied with the resources they received to optimize the exercises (81 and 86%, respectively). Moreover, both groups of students felt both activities improved their problem-solving capabilities at 79% (TBL) and 72% (PBL). Similarly, students’ reasoning capabilities were similar in both groups at 79% (TBL) and 71% (PBL). Both case groups (TBL) and control groups (PBL) were confident that they were ready to confront similar problems in real life at the respective levels of 77% (TBL) and 81% (PBL). In summary, the skills gained from both activities (TBL and PBL) resulted in a similar outcome, with no statistical significance—a *p*-value of 0.595 (Table 2).

#### 3.2.3 Attitude

The students in both the case group (TBL) and the control group (PBL) were actively engaged in our study, producing comparable outcomes of 83% for TBL and 86% for PBL, respectively. The control group (PBL) was motivated to engage in independent learning, attaining a rate of 86%, markedly superior to the case group’s rate (TBL) of 69%. Moreover, 86% of the control group (PBL) strongly concurred that they were permitted to collaborate as a team, in contrast to 73% of the case group (TBL) students who expressed agreement. Both groups actively participated in collecting the necessary information and data to resolve the issue at an equal rate of 79%. At 76 and 84%, respectively, both groups actively participated in modifying their original perceptions of the real-world issue. Both groups expressed satisfaction with the overall learning experience, with rates of 83% for TBL and 85%, respectively (Table 2).

**FIGURE 2:**
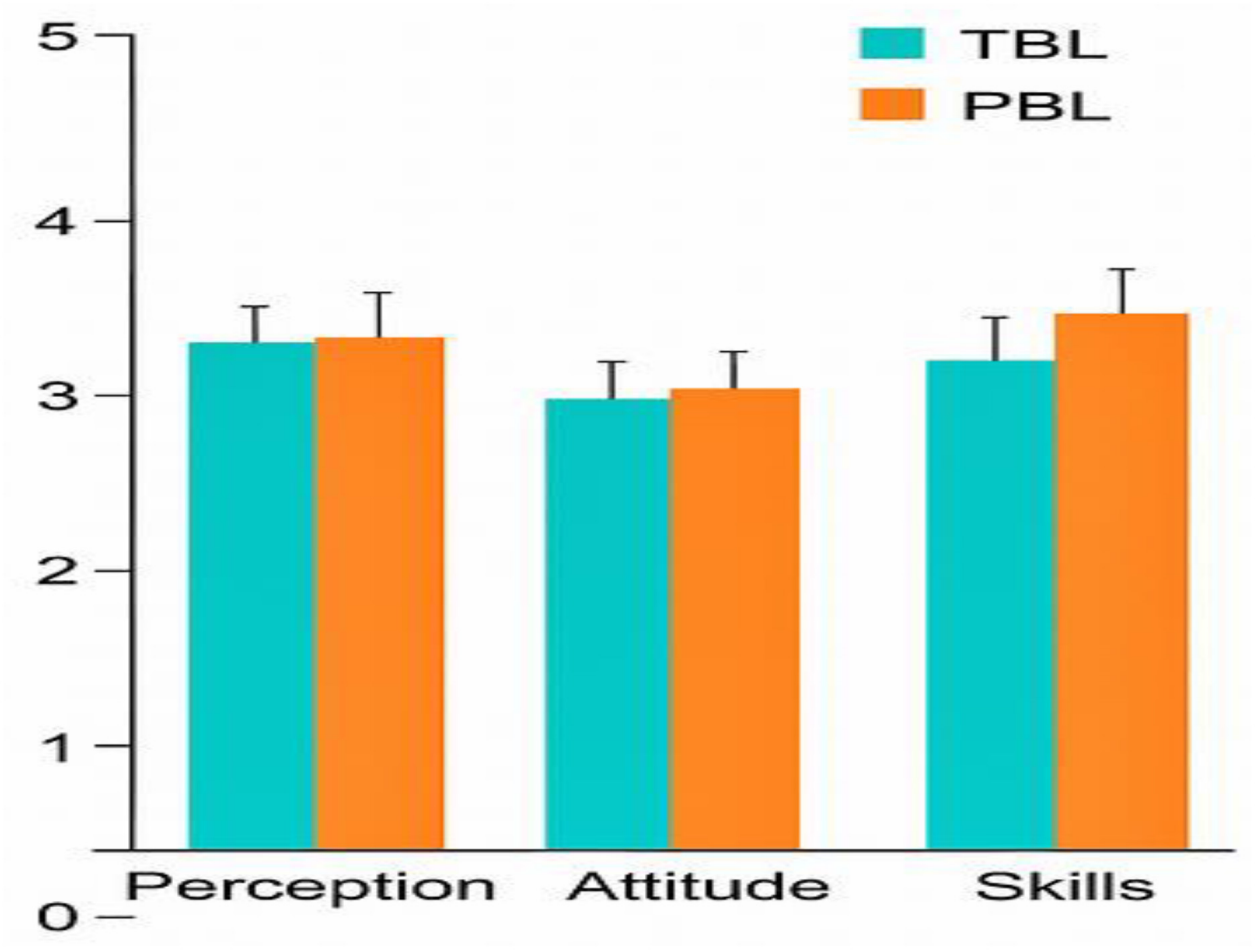
Telson questionnaire domains. TELSON questionnaire results comparing perception, attitude, and skills domains between TBL and PBL groups.

In conclusion, both groups exhibited no statistically significant difference in their attitudes toward either teaching style (*p* = 0.122) (Table 2; Figure 2).

### 3.3 Results from open-ended questions

A 41% of the case group (TBL) students responded to the question about the use of technology. The response rate in the control group (PBL) was 100%. Furthermore, 44% of the case group (TBL) responded regarding the description of the classrooms and the overall learning environment. Furthermore, 54% of the case group (TBL) students responded to the question by describing working with each other. 47% of the case group’s (TBL) students responded to the question based on their overall general experience.

### 3.4 Results of the pre- and post-summative tests

**FIGURE 3:**
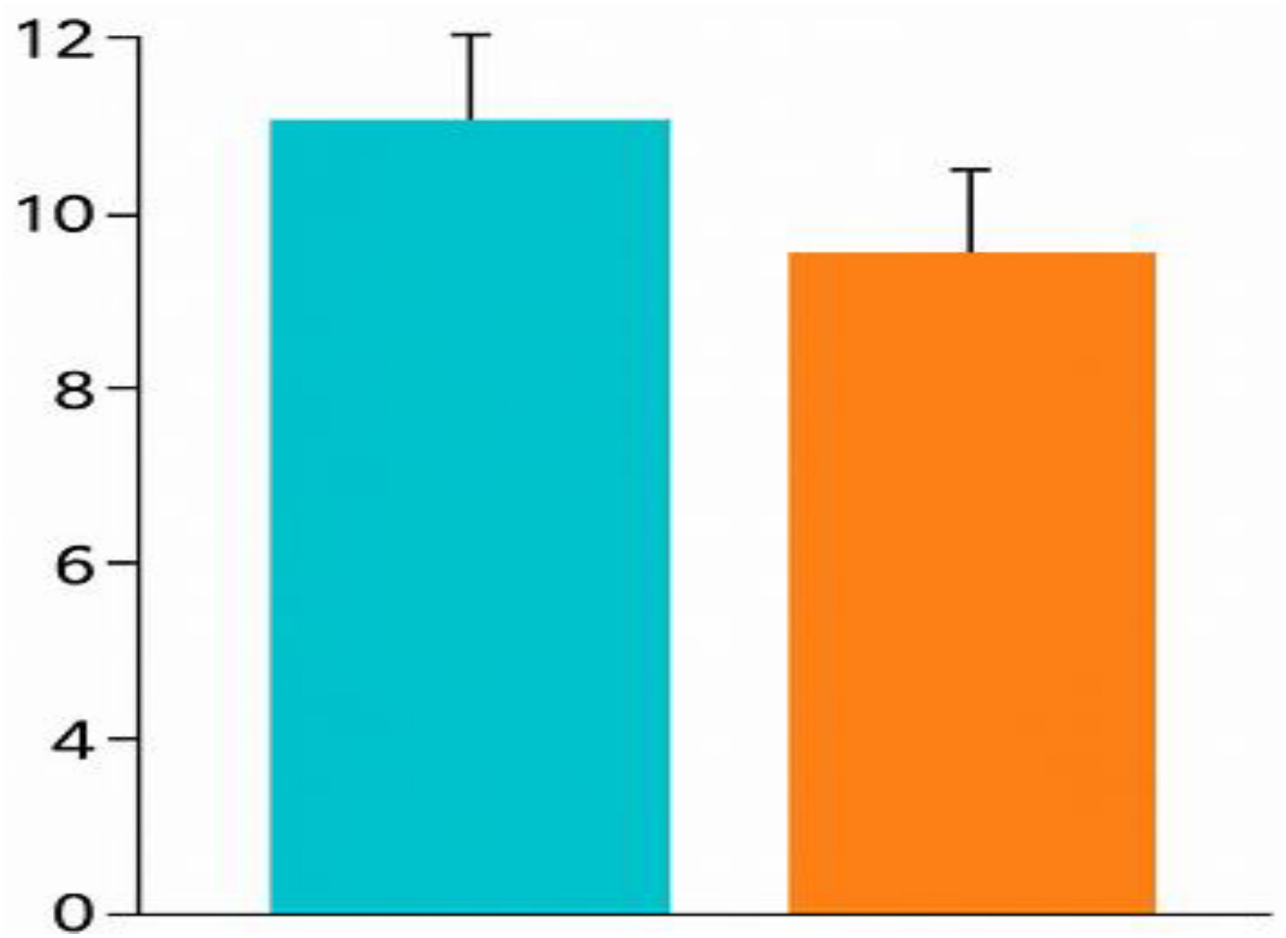
Summative test scores (TBL vs. PBL). Comparison of summative test scores between TBL and PBL groups. Error bars represent 95% confidence intervals.

The study revealed that students in the TBL group achieved an average score of 9.94 out of 14 on MCQs, with a standard deviation of 2.28. In contrast, the PBL group had an average score of 8.84 and a standard deviation of 2.24, with a *p*-value of 0.053 for both groups. The cumulative knowledge derived from summative/test results was not statistically significant for either group (Table 3; Figure 3).

**TABLE 3:** Summative assessment scores: TBL vs. PBL.

Group	Mean ± SD	Mean difference	*t*-value	*p*-value	Cohen’s *d*
TBL	9.94 ± 2.28	–	–	–	–
PBL	8.84 ± 2.24	-1.10	-1.97	0.053	-0.48

Independent-samples *t-*test comparing summative scores between groups. Cohen’s d indicates a small to moderate effect size.

**FIGURE 4:**
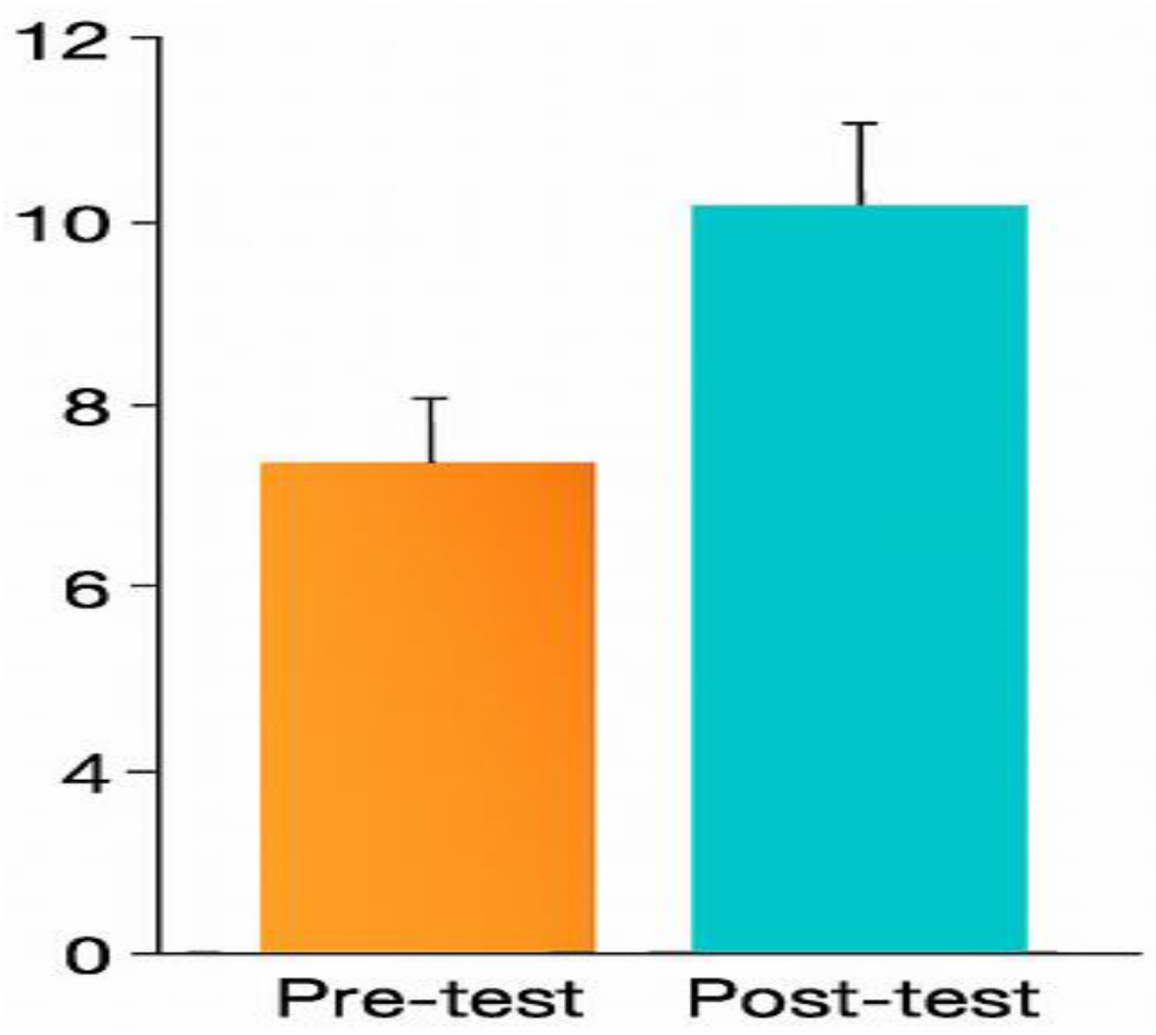
Pre- and post-test scores (TBL group). Pre- and post-test performance in the TBL group. Error bars represent 95% confidence intervals.

**FIGURE 5:**
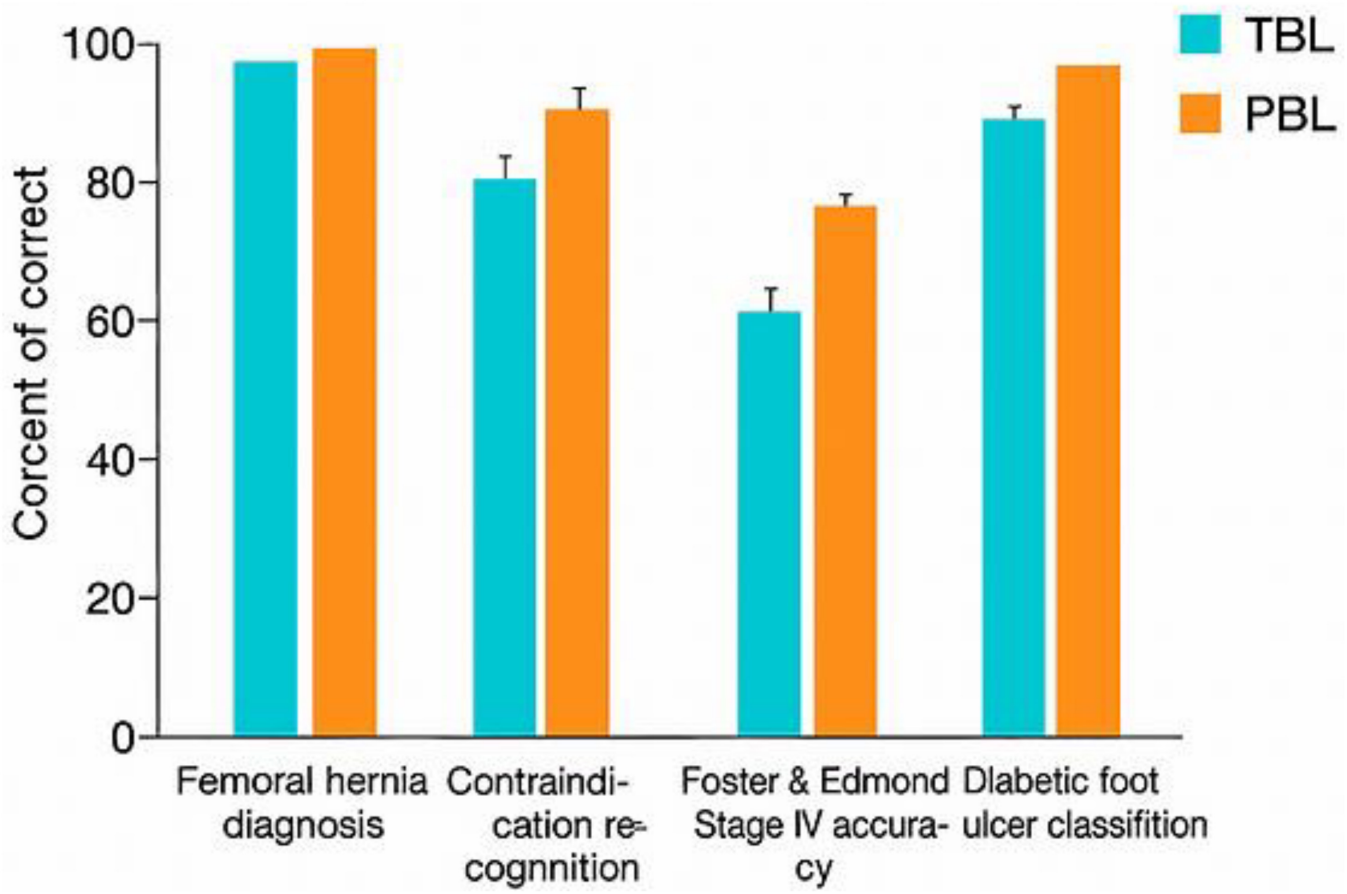
Clinical case performance. Item-level analysis of selected clinical cases comparing diagnostic, investigative, and staging accuracy between TBL and PBL groups.

In the TBL case-control experiment, the pre-class test mean score was 7.42 (*SD* = 2.50), while the post-test scores averaged 9.94 (*SD* = 2.35). This difference was statistically significant (*P* = 0.001), demonstrating the effectiveness of the TBL pedagogical loop in enhancing knowledge acquisition during the experience (Table 4; Figure 4). The outcomes of the summative assessment for both the TBS and BPL cohort groups were compiled according to several multiple-choice questions, as presented in Table 5 and Figure 5. Comparing pre-class test results with post-test results.

**TABLE 4:** Pre- and post-test scores in the TBL group.

Test type	Mean ± SD	*t*-value	*p*-value	Effect size (Cohen’s d)
Pre-test	7.42 ± 2.12	–	0.0001	–
Post-test	9.94 ± 2.28	4.21		0.52

Table 2 compares pre- and post-test scores within the TBL group. Paired *t*-test shows significant improvement with a moderate effect size.

**TABLE 5:** Clinical case performance by item at the summative test.

Case item	TBL correct (%)	PBL correct (%)
Femoral hernia diagnosis	85.3%	93.8%
Contraindication recognition	85.3%	100%
Erect chest X-ray reasoning	61.8%	31.3%
Foster and Edmond stage IV accuracy	85.3%	68.8%
Diabetic foot ulcer classification	88.2%	93.8%

Item-level analysis of selected clinical cases. PBL group performed better in diagnostic reasoning; TBL group showed stronger investigative and staging accuracy.

## 4 Discussion

The medical curriculum at the University of Tabuk is segmented into preclinical phases, including anatomy, physiology, and microbiology, where preclinical students are extensively engaged in Problem-Based Learning (PBL). Conversely, the clinical phase encompasses general surgery, medicine, obstetrics, and gynecology, where clinical students have had limited exposure to Team-Based Learning (TBL). The educators at our institution have extensive training and expertise in both TBP and PBL pedagogical approaches. TBL is renowned in medical education for its resource efficiency and student-centeredness; it was founded in 2001. A student-centered, organized TBL has basic design features and steps (22). TBL and PBL are “instructional activities involving students in doing things and contemplating what they are doing” (23), unlike standard lectures that cover one topic in one session. TBL involves large groups of 6–10 students in participatory learning. One instructor may effectively manage 20 teams using this way (24). TBL is an active method, but students learn in a controlled environment with instructor assistance (25). PBL students discover knowledge gaps and create self-study objectives. Unlike TBL, PBL demands more faculty and student self-directed study (26).

In our study, the post-test results indicated no superiority of either the TBL or BPL pedagogical strategy, as there was no statistical difference observed (*p*-value of 0.053 for both groups). Conversely, Smita Pakhmode indicated that “post-test results, obtained immediately following the intervention, revealed a significant learning gain in students utilizing both methods, with a statistically greater gain in TBL (*p* < 0.0001) relative to PBL” (3). “No statistically significant differences in knowledge retention were observed when the test was administered 2 months later in both groups.” It is important to note that our study did not assess the retention rate, as long-term retention tests were not conducted. Our study confirms the effectiveness of the loop (TBL) pedagogical strategy. The case groups utilizing TBL demonstrated enhanced knowledge acquisition through intergroup and intragroup discussions of clinical problems, as evidenced by the significant difference in pre- and post-test results (*P* = 0.001). We cannot underestimate the importance of having a clinically experienced teacher on site to implement iRAD, tRAD, immediate feedback, and intra-group discussion. Our study indicates that students in both case and control groups have experienced benefits from active engagement, such as enhanced problem-solving skills and overall student satisfaction. This conclusion is consistent with the findings of Sterpu et al. (21), which demonstrate that the implementation of TBL correlates with enhanced knowledge acquisition (*n* = 19, 39%), increased student engagement (*n* = 6, 12%), and greater student satisfaction (*n* = 31, 63%). Our study demonstrates that TBL enhances knowledge acquisition, as indicated by the comparison of pre- and post-test results, consistent with the findings of Mayel et al. (27) and Langer et al. (7). Despite numerous studies by Gong et al. (28) and Ackermann et al. (29) suggesting that the TBL has improved teamwork, our findings contradict this notion. Our study provided contrary results, with better teamwork in the control group (PBL). Conversely, TBL promotes a more scalable active-learning environment capable of accommodating bigger student populations without a corresponding increase in teacher time demands (4, 30). Our findings revealed that both the case group (TBL) and the control group (PBL) were satisfied with the overall experience, which is consistent with studies conducted by Krase et al. (31) and Thomas and Bowen (32), who discovered no differences in student satisfaction between TBL and non-TBL groups. Teaching effort and teachers’ attitudes toward TBL were the subject of numerous research studies, whereas the instructors stated that TBL was linked to a greater effort for class preparation than lecture-based instruction (12 h v. 5 h). However, we anticipated this effect because TBL was a recently developed technology. Additionally, the teachers said that using TBL made the classroom environment more active and engaging (33). However, our study did not statistically measure this factor because it fell outside our original targeting objectives. Few studies have assessed Team-Based Learning (TBL) in clinical stages, in contrast to more studies examining the role of TBL in basic science phases. Our institution extensively employs TBL methods during the preclinical phase of basic science, in contrast to minimal TBL methods during the clinical phases. This difference is explained by the fact that direct bedside teaching is the primary strategy for clinical instruction in various hospitals. Limited research has assessed the application of TBL in surgical disciplines, potentially due to the predominant instruction of surgical skills occurring in simulation environments, surgical wards, and operating theaters (34, 35). Clinical rotations are usually brief and do not allow students sufficient time to become familiar with TBL (36). Our result indicates that 91% of students were familiar with TBL, while the familiarity rate with PBL was 100%. Our study did not demonstrate the superiority of either method for measuring knowledge acquisition, despite the heavy use of PBL in our preclinical stages. Therefore, more long-term studies in our college would validate the superiority of one pedagogical strategy over the other. In the meantime, we recommend using hybrid teaching methods (traditional lectures, TBL, PBL, case-based discussion, seminars, tutorials, etc.) to evaluate our students, keeping in mind that one’s weakness can be compensated by another method. In 2006, problem-based learning (PBL) was incorporated into the medical program, introducing a well-established model of student-centered instruction in the curriculum. Increasing student enrolment from 36 first-year students in 2006 to 83 in 2025, coupled with limited teaching resources, has rendered this teaching model (PBL) unsustainable as the primary strategy for our expanding faculty. We recommend increasing the number of TBL sessions per batch during both the basic science and clinical phases, in conjunction with other pedagogical methods such as traditional lectures, seminars, tutorials, PBL, and case-based discussions (4). Furthermore, when compared to PBL, TBL retains the benefits of small group teaching and learning without requiring as many teachers (22). A notable limitation of this study is the lack of validation regarding whether all participating students engaged with the pre-class preparation material prior to attending class, as this aspect was not incorporated into our TELSON questionnaire. The absence of preparation has implications for team learning and performance, highlighting it as a critical area for further investigation (37). The literature indicates that administering the iRAT online before class enhances learning by decreasing the time required for the TBL experiment (38). However, we intentionally incorporated the iRAT into the overall exercise, despite its potential to extend the experiment’s duration from 2 to 4 h. We emphasized the inclusion of the iRAT as an integral component of TBL implementation with an expert, as our experience indicates that remote electronic questionnaires yield suboptimal response rates. The only way to guarantee that all respondents complete the iRAD and tRAD is through their actual presence in the class.

All the students used their laptops and mobile phones, as internet connectivity was stable and an electrical generator backup was available. One student mentioned that “’the iPad was used and the network was available in the building.”’ One student found using their iPad useful during the group discussion, stating, “The group discussion helped me learn how to read the question cautiously to understand what the best answer is.” The response rate in the control group (PBL) was 100%, with one student stating that “useful and advantageous technology helps us strengthen our knowledge.” One student stated that “the simulation room was comfortable” because the room was well air-conditioned and the walls had approved acoustic barriers. In contrast, one student in the control group (PBL) mentioned that “The environment was lovely, maybe somewhat cold.” Furthermore, 54% of the case group (TBL) students responded to the question by describing working with each other. In fact, one student mentioned that “I like it because we understand each other as a group.” Similar feedback was observed from the control group (PBL), as one student quoted that “I liked how we bounced ideas between each other and helped each other understand.” One student quoted that “it is my second time doing TBL, and I thoroughly enjoy this kind of learning; it makes me use my knowledge and add new information through the other options in the question.” In addition, all the students in the control group (PBL) had an encouraging experience, as one student mentioned that “the PBL classroom is a small-group, student-centered environment where students sit in a circle or U-shape to encourage discussion and collaboration.” The facilitator guides learning while students actively explore problems, share ideas, and access resources to support self-directed learning and solve the questions.

## 5 Conclusion

As a result, we showed that Team-Based Learning (TBL) and Problem-Based Learning (PBL) produced similar summative outcomes, with TBL providing stronger immediate knowledge acquisition. In this study, students overall valued two approaches more, with PBL being at least marginally preferred in the perception and attitude domains. The study’s findings indicate that each method possesses distinct advantages, demonstrating their mutually reinforcing roles within medical education. This study helps inform strategies of active learning to help provide problem-solving techniques, independence, and empowerment to future healthcare professionals, aligned with Vision 2030 priorities.

Despite that both TBL and PBL methodologies yielded comparable summative outcomes for both cohorts, the systemic implementation of TBL as a pedagogical tool can enhance students’ knowledge acquisition. Our study found that final-year MBBS students prefer both PBL and TBL in general, albeit that TBL enhances students’ knowledge acquisition, whereas PBL in perceptions and attitudes was marginally favored.

## 6 Recommendation

We advocate for the augmentation of TBL clinical sessions in general surgery, in conjunction with other conventional pedagogical methodologies, including PBL, due to the annual increase in student enrolment. The predominant instructional approach throughout the clinical stages at our medical faculty is bedside teaching in small groups. Consequently, most of our clinicians either refrain from utilizing TBL or lack familiarity with this educational approach. Consequently, we recommend promoting the involvement of newly recruited clinicians in the implementation of TBL through the organization of further workshops.

## 7 Limitation

Some important constraints are the lack of long-term retention assessments, the use of self-reported questionnaire data, and the lack of validation of pre-class prep. Though a readiness assurance test was included, a lack of standard validation might affect the results. However, the study’s limitations include its restriction to female cohorts and the possibility that cultural variables influenced learner views. Moreover, the research was conducted in a single institute, which reduces external validity. Another limitation is the short follow-up duration, which was not our objective, as well as the potential for response bias. Lastly, the input of experienced educators was not solicited.

## Data Availability

The raw data supporting the conclusions of this article will be made available by the authors, without undue reservation.
